# Light-Emitting Artificial Synapses for Neuromorphic Computing

**DOI:** 10.34133/2022/9786023

**Published:** 2022-09-23

**Authors:** Chen Zhu, Wen Huang, Wei Li, Xuegong Yu, Xing’ao Li

**Affiliations:** ^1^ College of Integrated Circuit Science and Engineering, Nanjing University of Posts and Telecommunications, Nanjing 210023, China; ^2^ New Energy Technology Engineering Laboratory of Jiangsu Province & School of Science, Nanjing University of Posts and Telecommunications, Nanjing 210023, China; ^3^ State Key Laboratory of Silicon Materials, Zhejiang University, Hangzhou 310027, China; ^4^ College of Electronic and Optical Engineering & College of Flexible Electronics (Future Technology), Nanjing University of Posts and Telecommunications, Nanjing 210023, China

## Abstract

As the key connecting points in the neuromorphic computing systems, synaptic devices have been investigated substantially in recent years. Developing optoelectronic synaptic devices with optical outputs is becoming attractive due to many benefits of optical signals in systems. Colloidal quantum dots (CQDs) are potential luminescent materials for information displays. Light-emitting diodes based on CQDs have become appealing candidates for optoelectronic synaptic devices. Moreover, light-emitting transistors exhibit great application potential in these synaptic devices. From this perspective, light-emitting artificial synapses were discussed on the basis of these structures in the devices. Their mechanisms, performance, and future development were analysed and prospected in detail.

## 1. Introduction

Neuromorphic computing, which is inspired by the human brain, is aimed at addressing challenges originating from the von Neumann bottleneck and promises to improve computing efficiency dramatically when processing complicated tasks [ 1]. As the core connection points in array neuromorphic computing systems, synaptic devices have been investigated substantially and have attracted much attention in recent years [ 2]. These devices are mainly divided into two categories. The first category is purely electronic with electrical signal input and electrical output, whereas the second is optoelectronic with optical signal input and electrical output [ 3]. Synaptic devices based on electrical output may face challenges, such as energy loss, sneak currents, and limited bandwidth, which significantly influence the application of neuromorphic computing. Thus, developing optoelectronic synaptic devices with optical output has attracted people’s interest. Compared with electrical signals, optical signals have the advantage of fast operation. Long-distance and one-to-many transmission could also be realised on the basis of optical signals in optoelectronic synaptic devices. Optical signals could realise spatial delivery, whereas sneak currents in the corresponding optoelectronic synaptic devices could be overcome by designing a self-rectifying structure [ 4], indicating strong anti-interference and avoiding energy loss capabilities on the basis of optoelectronic synaptic devices with optical output. Light-emitting diodes (LEDs) are semiconductor devices that could emit light, achieving a bandwidth of GHz magnitude [ 5, 6], demonstrating a much wider bandwidth than electrical signals. The electroluminescence (EL) effect of LEDs exhibits a memristive behaviour [ 7] and light-emission memory properties also exist in light-emitting transistors (LETs). These properties illustrate that light-emitting devices could be used as synaptic devices with optical output. Colloidal quantum dots (QDs) are solution-processed semiconducting nanocrystals that are compatible with a range of substrates [ 8]. These QDs have size-tuneable bandgaps with narrow band emission, which have been successfully used in LEDs and other light-emitting device designs. Naturally, QD light-emitting devices have generated considerable interest for their applications as optoelectronic synaptic devices in neuromorphic computing systems in recent years.

## 2. Progress and Future Development

**Figure 1:**
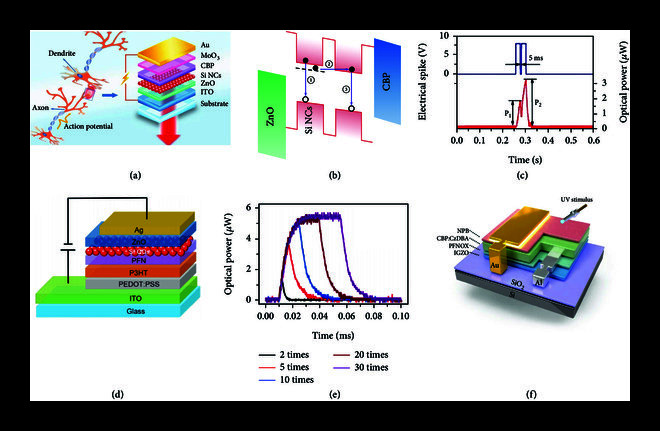
Demonstration of light-emitting devices in mimicking potentiation synaptic functions. (a) Schematic of a biological synapse and a light-emitting synaptic structure based on Si NCs. (b) Electron trapping process in the Si QDs. (c) Paired-pulse facilitation synaptic function based on the light-emitting synaptic device based on Si NCs. (a)–(c) are reproduced with permission from Ref. [ 10], copyright 2018 Elsevier Ltd. (d) Schematic of NIR light-emitting synaptic device based on Si NCs. (e) Synaptic plasticity in response to various spike numbers from 2 to 30. (d) and (e) are reproduced with permission from Ref. [ 13], copyright 2019, Science China Press and Springer-Verlag GmbH Germany, part of Springer Nature. (f) Device structure of long-afterglow organic light-emitting transistors. (f) is reproduced with permission from Ref. [ 14], copyright 2021 Wiley-VCH GmbH.

Silicon quantum dots (Si QDs) are potential luminescent materials for research on silicon-based light sources in optical interconnections [ 9]. Recently, Si QDs were used in a QLED-based synaptic device on the basis of the EL effect. This device was based on the multilayer structure of indium tin oxide (ITO)/ZnO/Si QDs/4,4 ′ -bis(N-carbazolyl)-1,1 ′ -biphenyl (CBP)/MoO _3_/Au, as demonstrated in Figure 1(a) [ 10]. In response to electrical spikes, electrons were injected into the conduction band minimum of the Si QDs from ITO (Figures 1(a) and 1(b)); holes were injected to the valance band maximum of the Si QDs from Au. This process led to the recombination between electrons and holes in the device, which corresponded to the EL with an external quantum efficiency (EQE) of ~3.0%. The emission wavelength was ~740 nm, which was consistent with a mean size of ~3.4 nm for Si QDs used in this synaptic device. Considering that dangling bonds exist in the surface of Si QDs, some electrons may be trapped by the dangling-bond-induced deep energy levels in Si QDs. Detrapping for these electrons is slow, leading to the long EL lifetime of about 20 ms for this QLED, which is in the decay time range of a signal in a biological synapse (1–10 ^4^ ms) [ 11]. On the basis of this property, the optical emission power, which represents the synaptic weight, could be modulated by the pulse duration time, pulse number, and frequency. Therefore, synaptic functions, such as paired-pulse facilitation (Figure 1(c)), transition from short-term potentiation (STP) to long-term potentiation (LTP), spike-rate-dependent potentiation, and symmetrical spike-timing-dependent plasticity (STDP), were successfully mimicked. In addition, logic functions, such as “AND,” “OR,” “NAND,” and “NOR,” based on these functions were realised in Si QD-based synaptic devices.

Near-infrared (NIR) emission (650–900 nm) is important in optical communication [ 12]; the loss of NIR light is low for its transmission through many technological systems. Therefore, developing NIR QLED for neuromorphic computing is crucial for the construction and application of neuromorphic computing systems. Photoluminescence spectra with a peak of approximately 850 nm for Si QDs were observed by increasing its size to a mean value of ~4.2 nm. These QDs that were incorporated into the structure of glass/ITO/poly(3,4-ethylenedioxythiophene):poly(styrenesulfonate) (PEDOT:PSS)/poly (3-hexylthiophene-2,5-diyl) (P3HT)/water-/alcohol-soluble polyelectrolyte poly [(9, 9-bis (3 ′ -(n,n-dimethylamino)propyl)-2,7-fluorene)-alt-2,7-(9,9-dioctylfluorene)] (PFN)/Si QDs/ZnO/Ag, as shown in Figure 1(d), can emit approximately 850 nm light with an EQE of 3.4% [ 13]. The dangling bonds on the surface of Si QDs could trap the electrons, leading to a 10 ms decay time for NIR emission. The optical power of NIR can thus be modulated through inputs of different electrical spike trains in this device, leading to a successful mimicking of important synaptic functions, such as STP and LTP in the NIR region based on this device (Figure 1(e)).

Recently, long-afterglow organic light-emitting transistors were fabricated on the basis of a bilayer structure of a TADF green-light-emitting organic material and an inorganic indium–gallium–zinc oxide (IGZO) semiconducting channel layer (Figure 1(f)) [ 14]. Upon UV irradiation, electrons present in excess on the IGZO surface absorb energy to be released into the bulk, which can finally drive the light-emission (525 nm) of the organic material. The decay time of the light emission based on the organic material is hundreds of seconds. This long-lived light-emission property is caused by the slow recombination kinetics of photogenerated electrons to oxygen vacancies in the IGZO channel layer. This property results in long-term plasticity of postsynaptic brightness based on the transistor structure, exhibiting great potential for its application in the light-emitting artificial synapses (LEASs). This property could ensure the recording of UV irradiation, demonstrating the enormous potential of synaptic devices for applications, such as visual UV micro-sized sensors.

**Figure 2:**
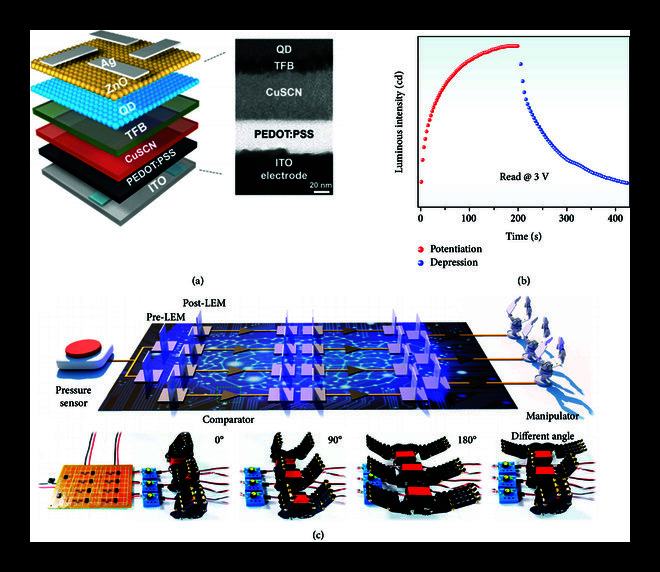
Demonstration of a light-emitting diode in mimicking potentiation and depression synaptic functions. (a) Schematic of a light-emitting synaptic device based on CdSe/ZnS QDs. (b) Light-emitting potentiation and depression properties. (c) Optoelectronic artificial efferent neural system used for controlling the manipulators intelligently based on the light-emitting CdSe/ZnS QDs synaptic device. (a)–(c) are reproduced with permission from Ref. [ 16], copyright 2021, American Chemical Society.

The realisation of mimicking a long-term depression (LTD) synaptic function can assure the mimicking of the most important learning mechanism, i.e., STDP, where synaptic efficacy was enhanced or depressed on the basis of the relative timing between presynaptic and postsynaptic spikes [ 15]. This function was crucial for the application of the constructed artificial neural networks. Chai et al. fabricated QLED-based synaptic devices based on the structure of ITO/PEDOS:PSS/cuprous thiocyanate (CuSCN)/poly[(9,9-dioctylfluorenyl-2,7-diyl)-co-(4,4 ′ -(N-(4-s-butylphenyl)diphenylamine))] (TFB)/CdSe/ZnS QDs/ZnO nanoparticles/Ag (Figure 2(a)) [ 16].

The current density–voltage–luminance relation of this device indicated a typical characteristic of a blue LED. In this stack, CuSCN and ZnO were used as the hole and electron transport layers, respectively; core/shell CdSe/ZnS QDs were used as an emissive layer. In response to electrical pulses, holes injected from ITO and electrons from Ag recombined in the emissive layer, resulting in blue light emission. This device exhibited a typical memristive behaviour on the emission property, which was attributed to the trapping of injected holes in the Cu SCN layer. If 200 electrical pulses were applied to this device, then the luminous intensity of this device gradually increased before reaching a saturation value. This process is the LTP process of synaptic plasticity. Interestingly, the LTD process can be realised if inverse electrical simulation pulses were applied (Figure 2(b)), which caused the hole release in the CuSCN layer.

This study is the first to display a QLED-based synaptic device that has LTP and LTD functions with the light signal output in response to external spikes, which utilise a hole transport layer as the trapping centre. The TFB layer can absorb blue light in the device. If a certain number of devices are integrated into a system, one pre-LED (equivalent to a presynaptic membrane) emits a blue light and the post-LED (a postsynaptic membrane) may receive the optical signal. Holes are generated in response to the optical spikes in the TFB layer and transferred to the CuSCN layer in post-LED. During this process, additional holes are generated in the TFB and trapped in the CuSCN layer under the illumination of high-input light intensity. The holes produce a built-in electric field that facilitates hole transport from the CuSCN layer to the TFB layer, leading to the improvement of conductivity for post-LED. An optoelectronic artificial efferent neural system can be constructed on the basis of this characteristic, which was used for emulating a biological reflex and controlling the manipulators intelligently (Figure 2(c)). In this system, the external mechanical signal can feed to the pre-LED through a pressure sensor. The pre-LED generates light signals that modulate the conductivity of post-LED, thus activating the manipulators, similar to the transmission between neurons in the human brain.

These LEAS-based synaptic devices have successfully mimicked various important synaptic functions with optical output, thus demonstrating some interesting applications as discussed above. These findings indicate the great potential of LEDs and LETs as synaptic devices in neuromorphic computing. However, the reported LEAS-based synaptic devices have some common problems [ 2]. First, the device area has a magnitude of approximately mm ^2^, and energy consumption $\left(>>\text{pJ}\right)$ for these devices is large. Thus, these devices cannot fulfil the requirement as synaptic devices in terms of device area (~nm ^2^) and energy consumption (~fJ) in the array systems. Second, the requirement on the linearity and dynamic range (>100) properties of synaptic weight (optical power) changes for synaptic devices is high, but these properties for the current light-emitting synapses remain insufficient for their applications, as shown in Figures 1(e) and 2(b). Third, the endurance properties were inferior for QLED-based synaptic devices, as seen from their LTP synaptic functions in the reference [ 9], whereas related research was missing in previous works [ 10, 13, 16]. These challenges impede the development of LEASs for neuromorphic computing.

## 3. Future Development

Light-emitting devices with high EQE efficiency and excellent stability must be sought to obtain high-performance LEASs for neuromorphic computing by considering the aspects of size, energy consumption, and endurance properties. Cd-based QDs may have the most potential in fabricating LEAS-based synaptic devices due to its excellent luminescence properties [ 4]. Further effort should be implemented to promote the application of Cd-based QDs in neuromorphic computing. Core/shell QDs must be conducted for Cd QD-based synaptic devices [ 16]. Intermediate shells, i.e., CdSe/CdS/ZnS and CdSe/ZnSe/ZnS, could be considered during this process, which may reduce the lattice-mismatch-induced trap states and improve efficiency. In addition, an appropriate surface ligand to the QDs is important for its properties. A short chain length and a strong binding to the surface of Cd QDs without developing defect states are the most important factors that must be considered. These characteristics are beneficial for the transporting properties of thin films and its efficiency. Inorganic halogen ions and organic phenylpropyl ligands have these properties and were suggested to be considered in the fabrication of LEAS-based synaptic devices. From a device engineering aspect, a large charge imbalance may arise from the different transport and injection capabilities of electrons and holes in these devices. Modifying the transport layers by doping or adding an external layer should be implemented to alleviate this disparity and thus improve efficiency. Furthermore, a highly uniform thin film calls for fabrication in the devices by modulating the size of QDs, thereby providing a foundation for the nanofabrication of the devices. Cd-free QDs, such as perovskite, LnP, ZnSe, CulnSe _2_, and Si QDs, are also interesting class materials for light emission. Light-emitting synaptic devices based on these materials should also be fabricated and investigated further. Material modification and device engineering should be implemented as in Cd QD-based synaptic devices to ensure the properties of Cd-free QD synaptic devices for neuromorphic computing.

On this basis, the nanoscale array structures of the devices must be considered because they are necessary for the construction of neuromorphic computing systems [ 17]. In this stage, the uniformity and thickness of the films in the devices need to be controlled precisely. Photolithography and electron beam lithography are proposed to fabricate these array systems on the basis of a specific pattern. These fabrication techniques may be combined with a selective electrophoretic deposition technique to achieve high-resolution, fast fabrication, and low-cost QD pattern devices [ 18]. These obtained devices with a nanoscale could increase light-emitting efficiency and decrease energy consumption and thus become favourable for the construction of array neuromorphic computing systems. In addition, developing all optical synaptic devices, which could completely avoid the challenges arising from electrical signals, is important. Various QDs that have a photoluminescence property from ultraviolet to infrared regions are encouraged to be used for fabricating LEAS-based synaptic devices. Furthermore, the modulation of emitting wavelengths through controlling size and doing in QDs is important. This can fulfil their requirement for vision sense and image recognition. In particular, infrared light emission is crucial to optical communication for neuromorphic computing. Thus, materials, such as PbS and Cu _2_SnS _3_ QDs, must be used to fabricate LEAS-based synaptic devices with infrared signal output. In addition, the encapsulation technique should be developed to promote the device endurance properties, which are crucial for the highly efficient application of neuromorphic computing systems.

Trapping on carriers in LEAS-based devices, which can regulate the recombination rate of carriers, is important for fulfilling their requirements as synaptic devices. This process can also ensure the device memory and accumulation properties, which are important for synaptic function mimicking. Trapping centres could be dangling bonds of QDs, defect states at the interface between emitting and transport layers, as well as in the transport layers, which could be introduced by annealing and doping amongst others. Meanwhile, these trapping centres may be the recombination centres and thus can influence emitting efficiency. Therefore, we must balance the response against trapping properties during this process, thus ensuring excellent light-emitting synaptic weight change properties by regulating the trapping centres. Meanwhile, light-emitting properties may exhibit volatile and nonvolatile characteristics based on different trapping capabilities. These characteristics in these devices should be investigated in detail for the construction of reservoir computing and other artificial neural network systems, respectively. Through these efforts, LEAS-based synaptic devices can be obtained with excellent performance, thus promoting the applications of neuromorphic computing in artificial intelligence, pattern recognition, and logic computing.
